# Leaf Extracts of *Mangifera indica* L. Inhibit Quorum Sensing – Regulated Production of Virulence Factors and Biofilm in Test Bacteria

**DOI:** 10.3389/fmicb.2017.00727

**Published:** 2017-04-24

**Authors:** Fohad M. Husain, Iqbal Ahmad, Abdullah S. Al-thubiani, Hussein H. Abulreesh, Ibrahim M. AlHazza, Farrukh Aqil

**Affiliations:** ^1^Department of Agricultural Microbiology, Faculty of Agricultural Sciences, Aligarh Muslim UniversityAligarh, India; ^2^Department of Food Science and Nutrition, College of Food and Agriculture Sciences, King Saud UniversityRiyadh, Saudi Arabia; ^3^Department of Biology, Faculty of Applied Science, Umm Al-Qura UniversityMakkah, Saudi Arabia; ^4^Department of Zoology, College of Science, King Saud UniversityRiyadh, Saudi Arabia; ^5^Department of Medicine and James Graham Brown Cancer Center, University of Louisville, LouisvilleKY, USA

**Keywords:** *Mangifera indica*, leaf extract, quorum sensing, biofilm inhibition, *C. elegans*, GC-MS, virulence factors

## Abstract

Quorum sensing (QS) is a global gene regulatory mechanism in bacteria for various traits including virulence factors. Disabling QS system with anti-infective agent is considered as a potential strategy to prevent bacterial infection. *Mangifera indica* L. (mango) has been shown to possess various biological activities including anti-QS. This study investigates the efficacy of leaf extracts on QS-regulated virulence factors and biofilm formation in Gram negative pathogens. Mango leaf (ML) extract was tested for QS inhibition and QS-regulated virulence factors using various indicator strains. It was further correlated with the biofilm inhibition and confirmed by electron microscopy. Phytochemical analysis was carried out using ultra performance liquid chromatography (UPLC) and gas chromatography–mass spectrometry (GC-MS) analysis. *In vitro* evaluation of anti-QS activity of ML extracts against *Chromobacterium violaceum* revealed promising dose-dependent interference in violacein production, by methanol extract. QS inhibitory activity is also demonstrated by reduction in elastase (76%), total protease (56%), pyocyanin (89%), chitinase (55%), exopolysaccharide production (58%) and swarming motility (74%) in *Pseudomonas aeruginosa* PAO1 at 800 μg/ml concentration. Biofilm formation by *P. aeruginosa* PAO1 and *Aeromonas hydrophila* WAF38 was reduced considerably (36–82%) over control. The inhibition of biofilm was also observed by scanning electron microscopy. Moreover, ML extracts significantly reduced mortality of *Caenorhabditis elegans* pre-infected with PAO1 at the tested concentration. Phytochemical analysis of active extracts revealed very high content of phenolics in methanol extract and a total of 14 compounds were detected by GC-MS and UPLC. These findings suggest that phytochemicals from the ML could provide bioactive anti-infective and needs further investigation to isolate and uncover their therapeutic efficacy.

## Introduction

Development of multidrug resistance in bacterial pathogens has created special problem for antibiotic therapy (MacPherson et al., 2009). Therefore, new drug target identification and development of new therapeutics to combat bacterial infection is currently needed. The attenuation of virulence factors and pathogenicity of bacteria through interfering quorum sensing (QS) is a possible alternative to killing or inhibiting growth of pathogenic bacteria (Fuqua et al., 2001; Rumbaugh et al., 2012; Ghosh et al., 2014). Many researchers have indicated that QS which regulates the expression of many virulence traits in pathogenic bacteria as an attractive anti-infective drug target (Smith and Iglewski, 2003; Rasmussen and Givskov, 2006). Autoinducer (AI), diffusible signaling molecules, regulate QS which allow bacteria to change their behavior in population density-dependent manner (Williams, 2007; Dandekar et al., 2012). AIs used by Gram negative bacteria are mainly *N*-Acyl homoserine lactones (AHLs) that play key roles in the virulence of pathogenic bacteria including *Pseudomonas aeruginosa* (Wang et al., 2009). *P. aeruginosa*, opportunistic pathogens, preferably infects the individuals with defective immune systems (Van Delden and Iglewski, 1998). The production of the majority of the virulence factors has been demonstrated to be regulated by one of the three QS systems, LasIR, RhlIR and PQS (McKnight et al., 2000; Smith and Iglewski, 2003; Jimenez et al., 2012).

*Pseudomonas* produces Las- and Rhl-dependent virulence factors and biofilm formation to facilitate the infection (Van Delden and Iglewski, 1998; Wang et al., 2009; Annapoorani et al., 2013). Bacterial biofilm development depends on release of extracellular polymeric compounds and QS mediated swimming and swarming motility (Pratt and Kolter, 1998; Jaisi et al., 2007). The bacteria in biofilm mode of growth become many fold tolerant to antibiotics than planktonic state of growth (Alhede et al., 2009). Biofilm formation inside the host resulted in successful establishment of pathogens and development of chronic infections in human (Annapoorani et al., 2013). The agents which obstruct bacterial QS and lead to the suppression of production of virulence factors are termed as QS inhibitors (Brackman et al., 2011). Such compounds were first reported in halogenated furanones of marine algae *Delisea pulchra* by Hentzer and Givskov (2003), but were found to be toxic and unstable.

Antibacterial activity of compounds from plants has been studied extensively. To obtain novel anti-QS agents, many workers have focused on natural products, because of the contribution in drug discovery. Recently, medicinal and food plants have been found to possess anti-QS property as reviewed by many workers (Musthafa et al., 2010; Husain and Ahmad, 2013b; Kalia, 2013; Zhao et al., 2013). Therefore, inhibition of QS has been envisioned to be the new target for developing anti-infective therapies because obstruction of QS will weaken the virulence of invading pathogens, making them more susceptible to the applied mode of treatment and facilitating easy clearance by host defense mechanism. However, discovery of therapeutically effective and safe anti-QS compounds is still in the stage of infancy and needs further exploration from natural products. In an ongoing screening program we have identified some potential anti-QS plants including *Mangifera indica* L.

*Mangifera indica* L. (Mango; family Anacardiaceae) has been shown to possess various medicinal properties (Shah et al., 2010). Polyphenolics, flavonoids and triterpenoids are the chief chemical constituents present in the plant. We have previously reported that leaf extract of *M. indica* demonstrated antibacterial, antifungal and antioxidant properties and we hypothesized that it may also be a potential source of anti-QS compounds. Therefore, in this manuscript we study the broad spectrum anti-QS and anti-biofilm properties of different fractions from ML extract against tester strains including *Chromobacterium violaceum*, *P. aeruginosa* PAO1 and *Aeromonas hydrophila*.

## Materials and Methods

### Bacterial Strains

*Chromobacterium violaceum* (12472), produces QS-regulated purple-colored pigment, violacein. *C. violaceum* (CVO26) is a Tn5 mutant strain derived from wild-type *C. violaceum* (CV31532) and it is unable to produce its own AHL, but retain the ability to respond against exogenous butanoyl (C_4_) and hexanoyl (C_6_) homoserine lactones (McLean et al., 1997). *P. aeruginosa* PAO1 is a pathogenic bacterium having QS-controlled virulence factors. These cultures were kindly provided by Dr. Robert J. C. Mclean, Texas State University, San Marcos, TX, USA. *Escherichia coli* MG4/pKDT17 is *E. coli* DH5α strain harboring plasmid pMG4/pKDT was kindly provided by Prof. Thomas K. Wood (Pennsylvania State University, USA). *A. hydrophila* WAF38 is an AHL producing our laboratory strain. *C. violaceum* 12472, *C. violaceum* CVO26, *A. hydrophila* WAF38 and *P. aeruginosa* PAO1 were maintained on Luria–Bertani (LB) broth at 28 and 37°C, respectively.

### Plant Material and Preparation of Extracts

*Mangifera indica* leaves were collected locally from the campus of Aligarh Muslim University, Aligarh, India. The mature dried leaves were finely ground and extract was prepared as described previously (Zahin et al., 2013, 2016). The extracts and fractions were dried by rotatory evaporator at 40°C followed by successive extraction of collected dried plant material with other solvents (benzene, ethyl acetate, acetone, methanol and ethanol). Each fraction was dried and store at 4°C. The yield of each fraction was determined. DMSO (0.1%) was used to reconstitute dry extracts for experimental purpose.

### Assay for Quorum Sensing Inhibition

Inhibition of QS by plant extracts was analyzed as described (McLean et al., 2004). Briefly, test organism *C. violaceum* (ATCC 12472; 10^6^ CFU/ml) was overlaid in 5 ml soft agar on the agar plates. Wells of 8 mm were made and loaded with 100 μl of solvent or plant extracts (100–1000 μg/ml). Fractions showing strong activity were repeated at lower concentrations using paper disk and tested by disk diffusion method. Inhibition of pigment production by the indicator strain around disk was considered as positive for QS interference. Similarly, assay was adopted with *C. violaceum* CVO26 with the addition of standardized 10 μM of C6-HSL (Sigma–Aldrich, St. Louis, MO, USA).

### MIC Determination

Broth dilution method was used to determine the minimum inhibitory concentration (MIC) of plant extracts. Tetrazolium salt (*p*-iodonitrotetrazolium violet) was used as an indicator of growth as described earlier (Ahmad and Aqil, 2007).

### Violacein Inhibition Assay

Production of violacein pigment by *C. violaceum* in the presence and absence of plant extract was analyzed by violacein extraction and quantified spectrophotometrically (Blosser and Gray, 2000). Briefly, 16–18 h (OD_600 nm_ = 0.1) grown culture was incubated in Erlenmeyer flasks containing Luria broth supplemented with C6-HSL (10 μM) in the absence and presence of plant extracts and incubated at 28°C for 24 h. For quantification, bacterial cells were collected and the pellet was dissolved in 1 ml DMSO. Cell debris was removed by centrifugation (13000 *g*; 10 min) and absorbance of soluble violacein was read at 585 nm using microplate reader (Thermo Scientific, Multiskan Ex, India). Percent inhibition of violacein production in the presence of plant extracts was measured as, [(OD of control - OD of treated)/OD of control] × 100. Simultaneously, cell viability of CVO26 strain was determined. Bacterial viable count was made by agar plate count method.

### Analysis of Bacterial Growth Curve

Effect of sub-MICs of ML extract on growth curve of PAO1 and WAF38 was determined. Bacterial culture was inoculated into LB broth (100 ml) with and without the extract. The flask was incubated at 37°C for 24 h. OD of the culture was monitored at 600 nm at interval of 2 h to plot the growth curve.

### Effect on Quorum Sensing Regulated Virulence Factors

#### Assay for *LasB* Elastolytic Activity

The method described by Adonizio et al. (2008) was adopted to determine elastolytic activity. Bacterial culture was treated with plant extracts for 16 h at 37°C. Both treated and untreated culture supernatant (100 μL each) was mixed with 900 μl of elastin congo red (ECR) buffer (100 mM Tris, 1 mM CaCl_2_, pH 7.5) containing 20 mg of elastin congo red (ECR, Sigma, USA). This mixture was incubated at 37°C on shaker incubator for 3 h followed by removal of insoluble ECR by centrifugation. The absorption of the congo red was determined by reading the supernatant at 495 nm and LB medium with or without ML extract was included as negative control.

#### Assay for Proteolytic Activity with Azocasein

The method described by Kessler et al. (1993) was adopted to determine proteolytic activity of cell-free supernatants of test bacteria (PAO1 and WAF38) cultured with and without ML extract at sub-MICs. Briefly, culture supernatants (150 μL each) were added to 1 ml of 0.3% azocasein (Sigma, USA) in 0.05 M Tris–HCl and 0.5 mM CaCl_2_ (pH 7.5) followed by incubation at 37°C for 15 min. The reaction was stopped by adding the trichloroacetic acid (l0%, 0.5 ml). After centrifugation the absorbance was read at 400 nm.

#### Assay for Pyocyanin Production

Method of Essar et al. (1990) was used to check the pyocyanin production. Briefly, culture supernatant (5 ml) of *P. aeruginosa* (PAO1) treated with or without ML extracts was first extracted with chloroform (3 ml) followed by re-extraction with 0.2 M HCl (1 ml). The solution was assayed for absorbance at 520 nm.

### Assay for Chitinase Activity

Chitinase activity was measured as described previously (Husain et al., 2013) adopting a modified chitin azure assay. The filter-sterilized culture supernatant was mixed in 2:1 with 0.1 M sodium citrate buffer (pH 4.8), containing chitin azure (0.5 mg/ml) followed by incubation on a shaker for 1 week at 37°C. The absorbance was recorded at 570 nm.

### Swarming Motility Assay

Swarming motility was determined as described earlier (Husain and Ahmad, 2013a). Briefly, the medium (1% tryptone, 0.5% NaCl and 0.3% agar) with or without various Sub MIC concentration of ML extracts were point inoculated with the test cultures (*P. aeruginosa* PAO1 and *C. violaceum*). After overnight incubation diameter of swarm was measured.

### Exopolysaccharide (EPS) Extraction and Estimation

Bacterial strains PAO1 and WAF38 were cultivated in the presence of ML extract and subsequently centrifuged to obtain supernatant and filter sterilized. Chilled absolute ethanol was added in the supernatant and left overnight to precipitate EPS at 4°C (Huston et al., 2004). The method of Dubois et al. (1951) was used to estimate sugar concentration.

### Assay for Biofilm Inhibition

The effect of ML at sub-MICs was determined using biofilm formation assay (O’Toole and Kolter, 1998). PAO1, WAF38 and CV 12472 cultures were grown overnight in the presence and absence of ML extract in microtitre plate. The biofilm was visualized after staining with crystal violet solution (0.1%). After removing planktonic cells by rinsing the microtitre plate, the dye was solubilized in the ethanol and quantified spectrophotometrically at 470 nm.

### *In Situ* Visualization of Biofilms

Scanning electron microscopy (SEM) was done to visualize the biofilm *in situ*. The method described for biofilm formation on glass cover slip was adopted as described earlier (Husain et al., 2013). Briefly, biofilms were grown for 24 h on glass coverslips using 12 well plate with plant extract treated and untreated cultures of PAO1. Non-adhered cells from the cover slips were removed by rinsing with with distilled water and processed for SEM using standard protocol.

### Effect on β-Galactosidase Activity

Method of Harjai et al. (2010) was used to determine the β-galactosidase reporter activity. Supernatants of PAO1 treated with mango extracts were extracted with ethyl acetate for QS signal molecules (AHLs). Then, 0.5 ml of ethyl acetate extract was incubated with 2 ml of reporter *E. coli* MG4 (pKDT17) strain at 30°C for 5 h in shaker-incubator. Pellet was collected (3200 × *g* for 15 min) and suspended in an equal volume of Z buffer (Na_2_HPO_4_, 0.06 M; NaH_2_PO_4_, 0.04 M; KCl, 0.01 M; MgSO_4_, 0.001 M; β-mercaptoethanol, 0.05 M; pH 7.0). To 1 ml of cell suspension, 1 ml of Z buffer, 200 μl chloroform and 100 μl of 0.1% SDS was added to lyse the cells, and 0.4 ml of *O*-nitrophenol-β-D-galactopyranoside (4 mg/ml in PBS) was added (Bhowmik and Marth, 1990). Reaction was stopped after the development of yellow color by adding 1 ml of 1 M Na_2_CO_3_. Absorbance was measured at 420 and 550 nm. Units of β-galactosidase were calculated as 1000 × OD_420 nm_ - (1.75 × OD_550 nm_) / time × volume× OD_600 nm_.

### Effect on *Caenorhabditis elegans* Survival

The method of Musthafa et al. (2012) was adopted to study *in vivo* efficiency of ML extract in *Caenorhabditis elegans* (*C. elegans*) nematode infection model. Briefly, the young adult nematodes were infected with PAO1 in the 24-well microtitre plate and incubated at 25°C for 12 h. After incubation, *C. elegans* from the wells were washed thrice with M9 buffer (KH_2_PO_4,_ 3 g; Na_2_HPO_4,_ 6 g; NaCl, 5 g; 1 M MgSO_4,_ 1 ml; and distilled water 1000 ml) to remove surface-bound bacteria. Around 10 infected worms were transferred to the wells of microtitre plate containing 10% LB broth in M9 buffer and incubated at 25°C with or without plant extract.

### Determination of Total Phenolic of Plant Extract

The total phenolic content of the ML extract was assayed by the method of Spanos and Wrolstad (1990), as described earlier (Zahin et al., 2010).

### Gas Chromatography–Mass Spectrometry (GC-MS) Analysis of Plant Extracts

The compositions of the ML extract was analyzed by gas chromatography as described earlier by Zahin et al. (2016).

### Ultra Performance Liquid Chromatography (UPLC) Analysis

The method describe earlier by Aqil et al. (2014) was adapted with little modification. Briefly, methanolic extracts of MLwas analyzed for the phenolics on a Shimadzu ultra performance liquid chromatography (UPLC) system comprised of two LC-20AD-XR pumps, SIL-20A-XR autosampler, and SPD-M20A photodiode array detector (PDA) controlled by Class VP software (ver 7.4, SP3) attached to a Shim-pack XR-ODS-II column (3.0 mm × 150 mm; 2.2 μ). A linear gradient of 3.5% phosphoric acid (solvent A) and acetonitrile (solvent B) with flow rate of 0.7 ml/min was used. In the gradient, solvent B was initially 15% for 2 min and increased to 20% by 3 min. The solvent B concentration was further increased to 60% from 3 to 10 min and held for 1 min and returned to 15% by 14 min. The chromatogram was collected from 200 to 600 nm and general phenolics were analyzed at 280 nm.

### Statistical Analysis

All experiments were performed in triplicate and the data obtained from the experiments were presented as mean values with or without standard deviation and the differences between control and test were analyzed using Student’s *t*-test.

## Results

Different extracts of ML were obtained in petroleum ether, benzene, ethyl acetate, acetone and methanol and tested for QS modulatory activity against *C. violaceum* (CV12472). The methanol fraction showed dose-dependent anti-QS activity being best effective without any growth inhibition at 400 μg/ml concentration (**Table 1**). At higher concentrations pigment inhibition, a determinant of anti-QS activity was accompanied by growth inhibition of CV12472. The ethyl acetate fraction also exhibited anti-QS activity but only at 1800 μg/ml concentration. Acetone fraction inhibited pigments at 400 and 800 μg/ml; however, it also inhibited bacterial growth at the same concentration. Petrol and benzene fractions showed no inhibition of QS at tested concentrations (200–1600 μg/ml). To assess the effect of ML fractions on QS-regulated functions MIC was determined. The MIC of the extract against CVO26 was 1000 μg/ml, and against *P. aeruginosa* PAO1 and *A. hydrophila* WAF38, the MIC was 2000 μg/ml.

**Table 1:** Inhibition of *C. violaceum* (CV12472) pigments by different fractions of *Mangifera indica*.

Fractions	Concentration (μg/ml)	Zone of inhibition (mm)		
		
		Total inhibition (*r*_1_)	Growth inhibition (*r*_2_)	Pigment inhibition (*r*_1_–*r*_2_)
Ethyl acetate	225	–	–	–


	450	–	–	–


	900	–	–	–


	1800	5	–	5


Acetone	100	–	–	–


	200	–	–	–


	400	18	15	3


	800	21	16	5


Methanol	200	–	–	–


	400	15	–	15


	800	21	2	19


	1600	25	8	17




*Data are the mean value of three replicate. Values are round off to nearest whole number. Petroleum ether and benzene fractions do not show any activity; –, No activity. Total inhibition = total zone of pigment inhibition including growth inhibition, if any.*

### Effects of ML Extract on QS-Regulated Virulence Factors/Traits

**FIGURE 1:**
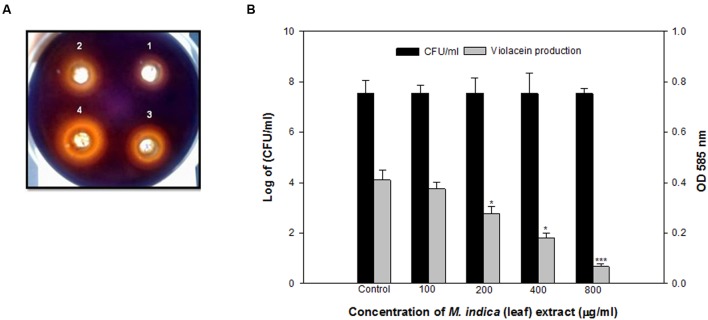
**(A)** Inhibition of violacein by methanol extract of *Mangifera indica* (leaf) in *C. violaceum* at the concentration of 200 (1), 400 (2), 800 (3), and 1600 (4) μg/ml. **(B)** Quantitative assessment of violacein inhibition in CVO26 at sub-inhibitory concentrations of *M. indica* (leaf) extract. All of the data are presented as mean ± standard deviation. ^∗^*p* ≤ 0.05, ^∗∗∗^*p* ≤ 0.001.

The methanol fraction demonstrated dose-dependent interference in QS activity as shown by reduced violacein production in CVO26 supplemented with synthetic AHL. It almost completely reduced the violacein production upto 83.6% at the concentration of 800 μg/ml. The effect was significant even at 400, 200, and 100 μg/ml concentrations with 55.6, 32.1, and 8.2% reductions, respectively (**Figures 1A,B**).

**Table 2** shows the dose-dependent effect of plant extracts on virulence factors in PAO1. Elastase activity decreased substantially at sub-inhibitory concentrations (100–800 μg/ml) of mango leaf (ML) extract with 17.6–76.2% reduction compared to the control. Similarly, a significant concentration-dependent decrease (43.8–56%) of total protease production was observed in the culture supernatant of *P. aeruginosa* at 200–800 μg/ml extract. Pre-treatment with extract (100, 200, 400, and 800 μg/ml) caused significant reduction of pyocyanin pigment (69.7, 80.2, 85.6, and 88.8%, respectively) in a dose-dependent manner (**Table 2**). Chitinase activity also decreased with ML extract but this decrease was significant (55.3%) only at 800 μg/ml conc. Similarly, a concentration-dependent decrease in the production of EPS was recorded in the treated culture of PAO1 compared to control. The extract exhibited statistically significant reduction of 50.2 and 58.3% in EPS at 400 and 800 μg/ml, respectively. In the present study, ML extract significantly reduced flagella-mediated swarming motility of *P. aeruginosa* PAO1 (25.2–73.7%) at tested sub-MICs (**Table 2** and **Figure 3**).

**Table 2:** Effect of *Mangifera indica* on inhibition of quorum sensing (QS) regulated virulence factors in *P. aeruginosa* PAO1.

Concentration (μg/ml)	Elastase activity^a^	Total protease^b^	Pyocyanin production^c^	Chitinase activity^d^	EPS production^e^	Swarming motility^f^	Biofilm formation^g^
Control	0.181 ± 0.04	1.091 ± 0.04	4.4 ± 0.34	0.141 ± 0.019	1.110 ± 0.02	67 ± 2.5	0.406 ± 0.04
100	0.149 ± 0.03 (17.6)	0.857 ± 0.03 (21.4)	1.33 ± 0.33 (69.7)^∗∗^	0.110 ± 0.02 (21.9)	0.678 ± 0.02 (38.9)	50 ± 2.0 (25.2)	0.256 ± 0.03 (36.9)
200	0.110 ± 0.02 (39.2)^∗^	0.613 ± 0.03 (43.8)^∗^	0.87 ± 0.20 (80.2)^∗∗^	0.09 ± 0.01 (36.1)	0.601 ± 0.02 (45.8)	34 ± 2.0 (48.9)^∗^	0.210 ± 0.02 (48.2)^∗^
400	0.090 ± 0.02 (50.2)^∗^	0.547 ± 0.02 (49.8)^∗^	0.63 ± 0.03 (85.6)^∗∗∗^	0.081 ± 0.01 (42.5)	0.552 ± 0.01 (50.2)^∗^	26 ± 1.5 (60.3)^∗^	0.179 ± 0.03 (55.9)^∗^
800	0.043 ± 0.02 (76.2)^∗∗^	0.479 ± 0.02 (56.0)^∗^	0.49 ± 0.02 (88.8)^∗∗∗^	0.063 ± 0.01 (55.3)^∗^	0.462 ± 0.02 (58.3)^∗^	17 ± 1.5 (73.7)^∗∗^	0.112 ± 0.02 (72.4)^∗∗^
Azithromycin (2 μg/ml)	0.048 ± 0.01	0.389 ± 0.02	0.2 ± 0.009	0.04 ± 0.01	0.34 ± 0.02	21.6 ± 2.3	0.095 ± 0.01


*^a^Elastase activity is expressed as the absorbance at 495 nm. ^b^Total protease activity is expressed as the absorbance at 600 nm_._^c^Pyocyanin concentrations were expressed as micrograms of pyocyanin produced per μg of total protein. ^d^Chitinase activity is expressed as the absorbance at 570 nm. ^e^EPS production is expressed as absorbance at 480 nm. ^f^Swarming motility is expressed as diameter of swarm in mm. ^g^Biofilm formation is expressed at 470 nm after incubation with crystal violet. Data represent mean and standard deviation of three independent experiments. Values in parentheses indicate percent reduction over control. ^∗^*p* ≤ 0.05, ^∗∗^*p* ≤ 0.005, ^∗∗∗^*p* ≤ 0.001.*

Effect of the methanol extract on virulence factors of *A. hydrophila* WAF38 is represented in **Table 3**. The extract demonstrated significant reduction in total protease activity (54–69%). EPS production in WAF38 strain was also reduced in a dose-dependent manner and significant decrease of 41.9, 52.2, and 59% was observed at 250, 500, and 1000 μg/ml concentration of the extract. Growth curve analysis showed that sub-MICs of *M. indica* did not cause significant change in the growth of *P. aeruginosa* PAO1 and *A. hydrophila* (Supplementary Figure S1)

**Table 3:** Effect of *Mangifera indica* leaf extract on inhibition of QS regulated virulence factors in *Aeromonas hydrophila* WAF-38.

Concentration (μg/ml)	Total protease^a^	EPS production^b^	Biofilm formation^c^
Control	0.589 ± 0.051	0.748 ± 0.021	0.226 ± 0.006
125	0.271 ± 0.015 (53.9)^∗^	0.661 ± 0.027 (11.6)	0.099 ± 0.004 (56.1)^∗^
250	0.223 ± 0.006 (62.1)^∗^	0.434 ± 0.009 (41.9)^∗^	0.087 ± 0.009 (61.5)^∗^
500	0.200 ± 0.010 (66.0)^∗∗^	0.357 ± 0.005 (52.2)^∗^	0.054 ± 0.006 (76.1)^∗∗^
1000	0.182 ± 0.004 (69.1)^∗∗^	0.306 ± 0.018 (59.0)^∗^	0.040 ± 0.003 (82.3)^∗∗∗^


*^a^Total protease activity is expressed as the absorbance at 600 nm_._^b^EPS production is expressed as absorbance at 480 nm_._^c^Biofilm formation is expressed at 470 nm after incubation with crystal violet. Data represent mean and standard deviation of three independent experiments. Values in parentheses indicate percent reduction over control. ^∗^*p* ≤ 0.05, ^∗∗^*p* ≤ 0.005, ^∗∗∗^*p* ≤ 0.001.*

#### Effect on Biofilm Formation

**FIGURE 2:**
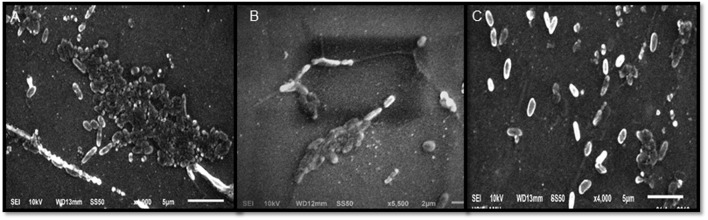
**Scanning electron microscopic (SEM) images for inhibition of biofilm of *Pseudomonas aeruginosa* PA01 at Sub minimum inhibitory concentrations.** Images shown the effect on biofilm formation at 400 μg/ml **(B)** and 800 μg/ml **(C)** concentration of *Mangifera indica* (methanol) extract compared to control **(A)**.

Sub-MICs were tested for biofilm inhibition in *P. aeruginosa* (PAO1) using crystal violet assay. Biofilm formation was significantly inhibited by 48, 56, and 72% at 200, 400, and 800 μg/ml concentrations, respectively (**Table 2**). SEM images also revealed reduction in the biofilm formation when treated with extracts (**Figure 2**). The impact of ML extract on WAF38 biofilm formation was also studied and a maximum reduction of 82.3% at 1000 μg/ml was recorded. At lower concentrations, there was also significant reduction in the range of 56–76% (**Table 3**).

**FIGURE 3:**
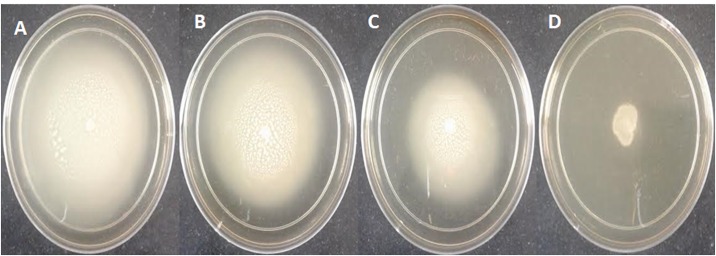
**Inhibition of swarming motility in *P. aeruginosa* PAO1 by sub-MICs of methanol extract of *M. indica* (leaf),** **(A)** Untreated control; **(B)** 200 μg/ml; **(C)** 400 μg/ml; **(D)** 800 μg/ml.

#### Effect on β-Galactosidase Activity

**FIGURE 4:**
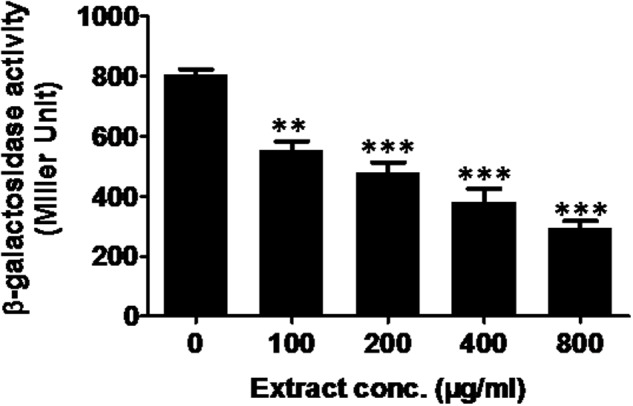
**Effect of *M. indica* (methanol) extract on β-galactosidase activity in *E. coli* MG4/pKDT17.** All of the data are presented as mean ± SD. ^∗∗^*p* ≤ 0.01, ^∗∗∗^*p* ≤ 0.001.

The las-controlled transmission was determined by inhibition of β-galactosidase activity. Mango extract at 800 μg/ml showed reduction in β-galactosidase in *E. coli* MG4/pKDT17 by 64% (**Figure 4**), suggesting direct inhibition of *las*-controlled transcription by ML bioactives. The effect was also significant at 200 and 400 μg/ml concentration.

#### Anti-infective Potential of Plant Extracts in *C. elegans* Nematode Model

**FIGURE 5:**
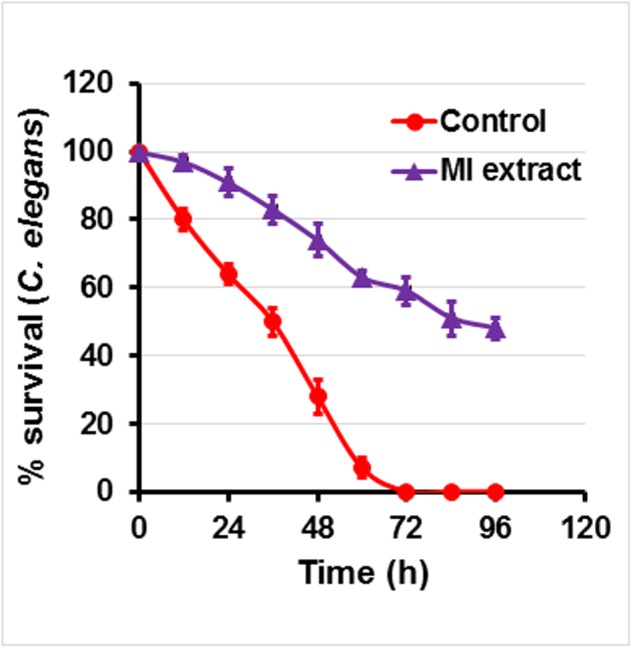
**Anti-infection potential of *M. indica* (methanol) extract on *P. aeruginosa* infected *C. elegans*.** Figure represents effect of mango extract (800 μg/ml) on the survival of *C. elegans*. Nematodes were pre-infected with *P. aeruginosa* and treated in the presence and absence of mango extract for 4 days. Graph represents the average of three independent experiments and SD.

In this model, *C. elegans* was infected with *P. aeruginosa* followed by treatment with ML extract. In the absence of extracts, 100% mortality of *C. elegans* was recorded after 72 h demonstrating the PAO1 pathogenicity. On the other hand, *C. elegans* pre-infected with PAO1 and maintained in the presence of ML extract (800 μg/ml) displayed an enhanced survival rate of 72% (**Figure 5**). The ML extracts could not exhibit significant mortality of *C. elegans* at the tested concentration.

### Phytochemical Analysis of Leaf Extract

**FIGURE 6:**
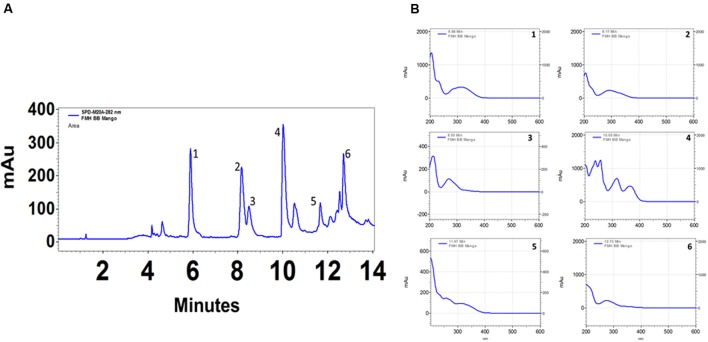
**HPLC profile of *Mangifera indica* (methanol) leaf extract.** Twenty microliter of extracts (1 mg/ml) were injected and analyzed using photo diode array detector on a C18 (250 mm × 4.6 mm ID × 5 μm) reverse phase column in a gradient of 3.5% aqueous phosphoric acid and acetonitrile. Chromatograms in **(A)** represent various polyphenolics detected at 280 nm. Chromatograms in the **(B)** represent analysis of different peaks as indicated in **(A)** in the range of 200–600 nm. mAu, milli absorbance units. For clarity of data, *y*-axis scales are different.

The total phenolic content (mg GAE/g) of extract was assayed by the Folin–Ciocalteu method and 497.6 gallic acid equivalents (mg/g) was recorded. Gas chromatography–mass spectrometry (GC-MS) analysis revealed 16 components in methanol fraction using a direct similarity search. The major compounds identified were pyrogallol (15.6%), benzoic acid, 4-hydroxy (12.09%), n-hexadecanoic acid (9.96%), 4H-pyran-4-one, 2,3-dihydro-3,5-dihydroxy-6-methyl (8.48%) as evident from the GC-MS spectra (**Table 4** and Supplementary Figures S2, S3). It is possible to extend the number of phytoconstiuents using chemometric techniques. We further analyzed the active methanolic fraction of ML with UPLC (**Figure 6**). The UPLC analysis revealed the presence of 7–8 major phytocompounds at different wavelength and distinct time of retention. The retention time of compounds was found between 6 and 13 min. The spectral analysis of major peaks revealed highest absorbance at around 270 nm suggesting their identity as phenolics. However, these peaks remained to be identified.

**Table 4:** Phytochemicals of *Mangifera indica* extract as identified by GC-MS analysis.

Peak no.	Components	Retention time	Area (%)
1.	1,3,5-Triazine-2,4,6-triamine	3.66	6.66
2.	1,2,3-Propanetriol, monoacetate	3.88	1.40
3.	4H-Pyran-4-one, 2,3-dihydro-3,5-dihydroxy-6-methyl	4.60	8.48
4.	2-Furancarboxaldehyde, 5-(hydroxymethyl)	5.90	4.72
5.	1,2,3-Benzenetriol	8.42	15.60
6.	Benzoic acid, 4-hydroxy	10.36	12.09
7.	1,2,3,4,5,6,7,8-Octahydro-2-naphthol, 4-methylene-2,5,5-trimethyl	11.61	1.03
8.	Tetradecanoic acid	12.63	1.72
9.	Pluchidiol	13.17	1.19
10.	Hexadecanoic acid, methyl ester	14.28	2.58
11.	n-Hexadecanoic acid	14.70	9.96
12.	9,12,15-Octadecatrienoic acid, methyl ester	15.99	1.71
13.	Phytol	16.11	2.24
14.	9,12,15-Octadecatrienoic acid, (Z,Z,Z)-	16.40	6.22
15.	Stigmast-5-en-3-ol, (3.beta.)-	29.47	2.32
16.	Lupeol	30.26	1.07


## Discussion

In many bacteria virulence factors are regulated through QS, a cell density-dependent global gene regulatory mechanism. Interference of QS circuits is considered as potential strategy to attenuate bacterial pathogenecity. In an ongoing program we have identified some potential plants with anti-QS activity in preliminary screening including *M. indica* L. (Zahin et al., 2010). In this study ML extract is subjected to sequential fractionation in different organic solvents and tested for QS interference by reduction in violacein production in *C. violaceum* 12472. Violacein production by *C. violaceum* is regulated by CviIR-dependent QS system (McLean et al., 2004). Therefore, any modulation of pigment in *C. violaceum* is considered direct evidence of QS interference.

The methanol fraction of ML apparently reduced the AHL-dependent pigment production, indicating interference of QS-controlled function. Further, interference of QS by the methanol fraction is also evident as it inhibited the violacein production in CVO26 without interfering the growth of *C. violaceum*. A similar effect on the violacein inhibition has been reported by *C. cyminum* and clove oil (Packiavathy et al., 2012; Husain et al., 2013). *C. violaceum* was used as the model for screening QS inhibitors because of the easy detection of the purple-colored pigmentation (violacein) in the indicator organism. Therefore, based on the violacein inhibition and lack of growth inhibition of the indicator organism CVO26, the methanol fraction was found the most active and thus further tested against *P. aeruginosa* PAO1 and *A. hydrophila* WAF38 to assess broad spectrum anti-QS activity because these organisms involve a different QS system than CVO26.

*Pseudomonas aeruginosa* secretes a range of QS-regulated virulence factors including elastase, protease and chitinase (Adonizio et al., 2008). The bacterial hydrolytic enzymes such as elastase and protease are known to affect the host cell proteins in the infected tissues and facilitate bacterial invasion and growth. In the present investigation, preincubation with the extract produced concentration-dependent inhibition of elastase and protease production. These data corroborated with the literature where, elastase activity, total proteolytic and chitinase activity of *P. aeruginosa* was decreased to varying levels (10–90%) by plant extracts and essential oils (Vattem et al., 2007; Husain and Ahmad, 2013b). Pyocyanin is another important virulence factor produced under QS-regulation. The role of pyocyanin is well documented in pathogenesis especially in cystic fibrosis (Winstanley and Fothergill, 2009). A significant dose-dependent decrease (*p* ≤ 0.001) was observed with ML extract in all strains, being maximum in PAO1 (88.8%). Similar reduction in pyocyanin production was recorded in extracts of *Terminalia chebula* (Sarabhai et al., 2013).

Quorum sensing mediated by AHLs are known to play significant role in biofilm formation. We found that the ML extract at Sub-MICs inhibited the biofilm biomass significantly (*P* ≤ 0.005) with no significant growth inhibition on PAO1. Sandasi et al. (2010) showed that surface adhesion followed by microcolony formation is promoted by surface conditioning.

Scanning electron microscopy images revealed ML extract efficiently reduced biofilm formed by bacteria which have further demonstrated that ML extract treatment reduces the biofilm strength. The possible interference in biofilm formation might be due to reducing surface adhesion or subsequent step of biofilm formation. The extract inhibiting QS is expected to adversely affect biofilm forming capacity (De Kievit et al., 2001). The results obtained in our study are in agreement with activity of extracts of *C. viminalis*, *Quercus virginiana*, *Tetrazygia bicolor* (Adonizio et al., 2008), *Lagerstroemia speciosa* (Singh et al., 2012), and *Sclerocarya birrea* (Sarkar et al., 2014).

Factors closely associated with biofilm formation of *P. aeruginosa* like swarming motility and EPS production were also evaluated in this study. Production of EPS is a prerequisite for the biofilm maturation (Watnick and Kolter, 1999), a QS regulated trait (Vu et al., 2009). Therefore, reduction in EPS production is due to QS interference by plant extracts (Abraham et al., 2011). It is hoped that plant extract significantly (*p* ≤ 0.05) reducing EPS will possibly reduce the resistance level of the pathogen in sessile mode. Treatment of PAO1 by plant extract reduces the EPS production. A dose-dependent decrease was observed in swarming motility of PAO1. It has been shown that flagellar-driven motility is required for surface attachment initiation in biofilm development (Pratt and Kolter, 1998). Therefore, inhibition in flagellar synthesis by the extracts would facilitate the reduced swarming migration. Thus, these extracts might have indirectly impacted biofilm formation by pathogens at least in part by disturbing AHL-mediated QS-system.

Virulence factors like exo-proteases production, EPS production and the biofilm formation are regulated by *ahyRI* QS system (Williams, 2007). ML extract demonstrated dose-dependent significant (*p* ≤ 0.005) reduction in the total protease and EPS production by *A. hydrophila* WAF38. Biofilm formation was also inhibited in a concentration-dependent manner by sub-MICs of extracts tested. The data obtained in our study for protease and biofilm inhibition indicated that the extract is potentially acting on the AhyRI system rendering the production of C4-HSL impaired. Similar findings against *Aeromonas* sp. were reported with chestnut honey and its extracts at 0.2 g/ml concentration, inhibiting biofilm up to 61% over control (Truchado et al., 2009).

The addition of ML extract decreased β-galactosidase luminescence in *E. coli* MG4/pKDT17 significantly (*p* ≤ 0.005) at sub-MICs. The results of the assay demonstrate reduction in AHL production under the effect of plant extract inhibits*las*-controlled transcription. It has been demonstrated that AHL is needed in combination with the *LasR* to achieve the maximal activation of the *lasB* gene, (Passador et al., 1993). Therefore, a critical global regulatory system is formed by *LasR* and AHL for the expression of *P. aeruginosa* virulence factors. The dependence of *lasB-lacZ* expression on AHL concentration has been reported (Pearson et al., 1994). Therefore, it is evident that reduction in the β-galactosidase activity in this study relates to reduce AHL levels through interference of *lasB* gene expression. Our results corroborates with the observations on *L. speciosa* fruit extract (Singh et al., 2012) and eugenol (Zhou et al., 2013).

Further, observation on the protection of *C. elegans* infected with *P. aeruginosa* PAO1 in the presence of ML extract provided a proof of the anti-virulence property. Our findings are in support of earlier works, which demonstrated similar activity of garlic extracts (Rasmussen and Givskov, 2006).

*Mangifera indica* L is rich in polyphenolic content and GC-MS analysis further confirmed the presence of an array of compounds. Pyrogallol was detected as the major compound which is known to exhibit antagonism against the QS signaling molecule AI-2 and virulence factors of *Vibrio* sp. (Brackman et al., 2008; Ni et al., 2008).

Inhibition of multiple traits regulated by QS by ML extract indicated its broad-spectrum activity through the QS interference possibly at multiple targets. Various compounds present in the extract might also influence QS by indirectly acting on the other primary target linked to QS functioning. The compounds like trifluoromethyl ketones, phenothiazine and phenothiazine thioridazine that inhibit proton motive force linked activities such as motility of Gram negative bacteria, and also inhibitor of the proton motive force-dependent efflux pump system of bacteria and QS system (Varga et al., 2012). The possible role of various synthetic and natural compounds has been reported as efflux pump inhibitor of Gram negative bacteria and QS interference (Amaral and Molnar, 2012). Therefore, presence of such compounds in the plant extracts may also influence the anti-QS activity through the efflux pump inhibition and needs further investigation. On the other hand, pyrogallol detected in this study is known to inhibit QS through generation of H_2_O_2_ that somehow interfere with the expression of bioluminescence in *Vibrio* (Defoirdt et al., 2013).

Our data indicated that ML extracts and its active constituents as a potential candidate for exploiting as anti-infective agent in modern phytomedicine. Further identifying and exploring active compounds as novel anti-QS agent that effectively inhibit virulence and demonstrate the therapeutic utility against drug resistant bacteria are needed.

## Author Contributions

FMH carried out all screening experiments and characterization of their anti QS properties. FA designed and carried out UPLC studied. AA-t, HHA, and IMA contributed to phytochemical analysis of GC result and contributed in the writing and revision of the manuscript. IA designed and supervised all the work. All authors read and approved the final version of the manuscript.

## Conflict of Interest Statement

The authors declare that the research was conducted in the absence of any commercial or financial relationships that could be construed as a potential conflict of interest.

## References

[B1] Abraham S. V. Palani A. Ramaswamy B. R. Shunmugiah K. P. Arumugam V. R. (2011). Antiquorum sensing and antibiofilm potential of *Capparis spinosa*. *Arch. Med. Res.* 42 658–668. 10.1016/j.arcmed.2011.12.002 22222491

[B2] Adonizio A. Kong K. F. Mathee K. (2008). Inhibition of quorum sensing-controlled virulence factor production in *Pseudomonas aeruginosa* by south Florida plant extracts. *Antimicrob. Agents Chemother.* 52 198–203. 10.1128/AAC.00612-07 17938186 PMC2223872

[B3] Ahmad I. Aqil F. (2007). In vitro efficacy of bioactive extracts of 15 medicinal plants against ES beta L-producing multidrug-resistant enteric bacteria. *Microbiol. Res.* 162 264–275. 10.1016/j.micres.2006.06.010 16875811

[B4] Alhede M. Bjarnsholt T. Jensen P. O. Phipps R. K. Moser C. Christophersen L. (2009). *Pseudomonas aeruginosa* recognizes and responds aggressively to the presence of polymorphonuclear leukocytes. *Microbiology* 155 3500–3508. 10.1099/mic.0.031443-0 19643762

[B5] Amaral L. Molnar J. (2012). Inhibitors of efflux pumps of Gram-negative bacteria inhibit Quorum Sensing. *Open J. Pharmacol.* 2 2–15.

[B6] Annapoorani A. Kalpana B. Musthafa K. S. Pandian S. K. Ravi A. V. (2013). Antipathogenic potential of *Rhizophora* spp. against the quorum sensing mediated virulence factors production in drug resistant *Pseudomonas aeruginosa*. *Phytomedicine* 20 956–963. 10.1016/j.phymed.2013.04.011 23746758

[B7] Aqil F. Vadhanam M. V. Jeyabalan J. Cai J. Singh I. P. Gupta R. C. (2014). Detection of anthocyanins/anthocyanidins in animal tissues. *J. Agric. Food Chem.* 62 3912–3918. 10.1021/jf500467b 24650213 PMC4334289

[B8] Bhowmik T. Marth E. H. (1990). β-Galactosidase of *Pediococcus* species: induction, purification and partial characterization. *Appl. Microbiol. Biotechnol.* 33 317–323. 10.1007/BF00164529

[B9] Blosser R. S. Gray K. M. (2000). Extraction of violacein from *Chromobacterium violaceum* provides a new quantitative bioassay for *N*-acyl homoserine lactone autoinducers. *J. Microbiol. Methods* 40 47–55. 10.1016/S0167-7012(99)00136-0 10739342

[B10] Brackman G. Cos P. Maes L. Nelis H. J. Coenye T. (2011). Quorum sensing inhibitors increase the susceptibility of bacterial biofilms to antibiotics in vitro and in vivo. *Antimicrob. Agents Chemother.* 55 2655–2661. 10.1128/AAC.00045-11 21422204 PMC3101409

[B11] Brackman G. Defoirdt T. Miyamoto C. Bossier P. Van Calenbergh S. Nelis H. (2008). Cinnamaldehyde and cinnamaldehyde derivatives reduce virulence in *Vibrio* spp. by decreasing the DNA-binding activity of the quorum sensing response regulator LuxR. *BMC Microbiol.* 8:149 10.1186/1471-2180-8-149 PMC2551610 18793453

[B12] Dandekar A. A. Chugani S. Greenberg E. P. (2012). Bacterial quorum sensing and metabolic incentives to cooperate. *Science* 338 264–266. 10.1126/science.1227289 23066081 PMC3587168

[B13] De Kievit T. R. Gillis R. Marx S. Brown C. Iglewski B. H. (2001). Quorum-sensing genes in *Pseudomonas aeruginosa* biofilms: their role and expression patterns. *Appl. Environ. Microbiol.* 67 1865–1873. 10.1128/AEM.67.4.1865-1873.2001 11282644 PMC92808

[B14] Defoirdt T. Pande G. S. J. Baruah K. Bossier P. (2013). The apparent quorum-sensing inhibitory activity of pyrogallol is a side effect of peroxide production. *Antimicrob. Agents Chemother.* 57 2870–2873. 10.1128/AAC.00401-13 23545532 PMC3716134

[B15] Dubois M. Gilles K. Hamilton J. K. Rebers P. A. Smith F. (1951). A colorimetric method for the determination of sugars. *Nature* 168:167 10.1038/168167a0 14875032

[B16] Essar D. W. Eberly L. Hadero A. Crawford I. P. (1990). Identification and characterization of genes for a second anthranilate synthase in *Pseudomonas aeruginosa*: interchangeability of the two anthranilate synthases and evolutionary implications. *J. Bacteriol.* 172 884–900. 10.1128/jb.172.2.884-900.1990 2153661 PMC208517

[B17] Fuqua C. Parsek M. R. Greenberg E. P. (2001). Regulation of gene expression by cell-to-cell communication: acyl-homoserine lactone quorum sensing. *Annu. Rev. Genet.* 35 439–468. 10.1146/annurev.genet.35.102401.090913 11700290

[B18] Ghosh R. Tiwary B. K. Kumar A. Chakraborty R. (2014). Guava leaf extract inhibits quorum-sensing and *Chromobacterium violaceum* induced lysis of human hepatoma cells: whole transcriptome analysis reveals differential gene expression. *PLoS ONE* 9:e107703 10.1371/journal.pone.0107703 PMC4167859 25229331

[B19] Harjai K. Kumar R. Singh S. (2010). Garlic blocks quorum sensing and attenuates the virulence of *Pseudomonas aeruginosa*. *FEMS Immunol. Med. Microbiol.* 58 161–168. 10.1111/j.1574-695X.2009.00614.x 19878318

[B20] Hentzer M. Givskov M. (2003). Pharmacological inhibition of quorum sensing for the treatment of chronic bacterial infections. *J. Clin. Invest.* 112 1300–1307. 10.1172/JCI20074 14597754 PMC228474

[B21] Husain F. M. Ahmad I. (2013a). Doxycycline interferes with quorum sensing-mediated virulence factors and biofilm formation in gram-negative bacteria. *World J. Microbiol. Biotechnol.* 29 949–957. 10.1007/s11274-013-1252-1 23299903

[B22] Husain F. M. Ahmad I. (2013b). Quorum sensing inhibitors from natural products as potential novel antiinfective agents. *Drug Future* 38 691–706. 10.1358/dof.2013.038.10.2025393

[B23] Husain F. M. Ahmad I. Asif M. Tahseen Q. (2013). Influence of clove oil on certain quorum-sensing-regulated functions and biofilm of *Pseudomonas aeruginosa* and *Aeromonas hydrophila*. *J. Biosci.* 38 835–844. 10.1007/s12038-013-9385-9 24296886

[B24] Huston A. L. Methe B. Deming J. W. (2004). Purification, characterization, and sequencing of an extracellular cold-active aminopeptidase produced by marine psychrophile *Colwellia psychrerythraea* strain 34H. *Appl. Environ. Microbiol.* 70 3321–3328. 10.1128/AEM.70.6.3321-3328.2004 15184127 PMC427748

[B25] Jaisi D. P. Dong H. L. Kim J. He Z. Q. Morton J. P. (2007). Nontronite particle aggregation induced by microbial Fe(III) reduction and exopolysaccharide production. *Clay Clay Miner.* 55 96–107. 10.1346/CCMN.2007.0550108

[B26] Jimenez P. N. Koch G. Thompson J. A. Xavier K. B. Cool R. H. Quax W. J. (2012). The multiple signaling systems regulating virulence in *Pseudomonas aeruginosa*. *Microbiol. Mol. Biol. Rev.* 76 46–65. 10.1128/MMBR.05007-11 22390972 PMC3294424

[B27] Kalia V. C. (2013). Quorum sensing inhibitors: an overview. *Biotechnol. Adv.* 31 224–245. 10.1016/j.biotechadv.2012.10.004 23142623

[B28] Kessler E. Safrin M. Olson J. C. Ohman D. E. (1993). Secreted LasA of *Pseudomonas aeruginosa* is a staphylolytic protease. *J. Biol. Chem.* 268 7503–7508. 8463280

[B29] MacPherson D. W. Gushulak B. D. Baine W. B. Bala S. Gubbins P. O. Holtom P. (2009). Population mobility, globalization, and antimicrobial drug resistance. *Emerg. Infect. Dis.* 15 1727–1732. 10.3201/eid1511.090419 19891858 PMC2857230

[B30] McKnight S. L. Iglewski B. H. Pesci E. C. (2000). The *Pseudomonas* quinolone signal regulates rhl quorum sensing in *Pseudomonas aeruginosa*. *J. Bacteriol.* 182 2702–2708. 10.1128/JB.182.10.2702-2708.2000 10781536 PMC101972

[B31] McLean R. J. Whiteley M. Stickler D. J. Fuqua W. C. (1997). Evidence of autoinducer activity in naturally occurring biofilms. *FEMS Microbiol. Lett.* 154 259–263. 10.1111/j.1574-6968.1997.tb12653.x 9311122

[B32] McLean R. J. C. Pierson L. S. Fuqua C. (2004). A simple screening protocol for the identification of quorum signal antagonists. *J. Microbiol. Methods* 58 351–360. 10.1016/j.mimet.2004.04.016 15279939

[B33] Musthafa K. S. Balamurugan K. Pandian S. K. Ravi A. V. (2012). 25-Piperazinedione inhibits quorum sensing-dependent factor production in *Pseudomonas aeruginosa* PAO1. *J. Basic Microbiol.* 52 679–686. 10.1002/jobm.201100292 22359266

[B34] Musthafa K. S. Ravi A. V. Annapoorani A. Packiavathy I. S. V. Pandian S. K. (2010). Evaluation of anti-quorum-sensing activity of edible plants and fruits through inhibition of the N-Acyl-homoserine lactone system in *Chromobacterium violaceum* and *Pseudomonas aeruginosa*. *Chemotherapy* 56 333–339. 10.1159/000320185 20720417

[B35] Ni N. T. Choudhary G. Li M. Y. Wang B. H. (2008). Pyrogallol and its analogs can antagonize bacterial quorum sensing in *Vibrio harveyi*. *Bioorg. Med. Chem. Lett.* 18 1567–1572. 10.1016/j.bmcl.2008.01.081 18262415

[B36] O’Toole G. A. Kolter R. (1998). Initiation of biofilm formation in *Pseudomonas fluorescens* WCS365 proceeds via multiple, pathways convergent signalling: a genetic analysis. *Mol. Microbiol.* 28 449–461. 10.1046/j.1365-2958.1998.00797.x 9632250

[B37] Packiavathy I. A. S. V. Agilandeswari P. Musthafa K. S. Pandian S. K. Ravi A. V. (2012). Antibiofilm and quorum sensing inhibitory potential of *Cuminum cyminum* and its secondary metabolite methyl eugenol against Gram negative bacterial pathogens. *Food Res. Int.* 45 85–92. 10.1016/j.foodres.2011.10.022

[B38] Passador L. Cook J. M. Gambello M. J. Rust L. Iglewski B. H. (1993). Expression of *Pseudomonas aeruginosa* virulence genes requires cell-to-cell communication. *Science* 260 1127–1130. 10.1126/science.8493556 8493556

[B39] Pearson J. P. Gray K. M. Passador L. Tucker K. D. Eberhard A. Iglewski B. H. (1994). Structure of the autoinducer required for expression of *Pseudomonas aeruginosa* virulence genes. *Proc. Natl. Acad. Sci. U.S.A.* 91 197–201. 10.1073/pnas.91.1.197 8278364 PMC42913

[B40] Pratt L. A. Kolter R. (1998). Genetic analysis of *Escherichia coli* biofilm formation: roles of flagella, motility, chemotaxis and type I pili. *Mol. Microbiol.* 30 285–293. 10.1046/j.1365-2958.1998.01061.x 9791174

[B41] Rasmussen T. B. Givskov M. (2006). Quorum sensing inhibitors: a bargain of effects. *Microbiology* 152 895–904. 10.1099/mic.0.28601-0 16549654

[B42] Rumbaugh K. P. Trivedi U. Watters C. Burton-Chellew M. N. Diggle S. P. West S. A. (2012). Kin selection, quorum sensing and virulence in pathogenic bacteria. *Proc. Biol. Sci.* 279 3584–3588. 10.1098/rspb.2012.0843 22648154 PMC3396913

[B43] Sandasi M. Leonard C. M. Viljoen A. M. (2010). The in vitro antibiofilm activity of selected culinary herbs and medicinal plants against *Listeria monocytogenes*. *Lett. Appl. Microbiol.* 50 30–35. 10.1111/j.1472-765X.2009.02747.x 19874481

[B44] Sarabhai S. Sharma P. Capalash N. (2013). Ellagic acid derivatives from *Terminalia chebula* Retz. downregulate the expression of quorum sensing genes to attenuate *Pseudomonas aeruginosa* PAO1 virulence. *PLoS ONE* 8:e53441 10.1371/journal.pone.0053441 PMC3539995 23320085

[B45] Sarkar R. Chaudhary S. K. Sharma A. Yadav K. K. Nema N. K. Sekhoacha M. (2014). Anti-biofilm activity of Marula – a study with the standardized bark extract. *J. Ethnopharmacol.* 154 170–175. 10.1016/j.jep.2014.03.067 24742751

[B46] Shah K. A. Patel M. B. Patel R. J. Parmar P. K. (2010). *Mangifera indica* (mango). *Pharmacog. Rev.* 4 42–48. 10.4103/0973-7847.65325 PMC3249901 22228940

[B47] Singh B. N. Singh H. B. Singh A. Singh B. R. Mishra A. Nautiyal C. S. (2012). *Lagerstroemia speciosa* fruit extract modulates quorum sensing-controlled virulence factor production and biofilm formation in *Pseudomonas aeruginosa*. *Microbiology* 158 529–538. 10.1099/mic.0.052985-0 22117007

[B48] Smith R. S. Iglewski B. H. (2003). *P. aeruginosa* quorum-sensing systems and virulence. *Curr. Opin. Microbiol.* 6 56–60. 10.1016/S1369-5274(03)00008-0 12615220

[B49] Spanos G. A. Wrolstad R. E. (1990). Influence of processing and storage on the phenolic composition of Thompson Seedless grape juice. *J. Agric. Food Chem.* 38 1565–1571. 10.1021/jf00097a030

[B50] Truchado P. Gil-Izquierdo A. Tomas-Barberan F. Allende A. (2009). Inhibition by chestnut honey of N-Acyl-L-homoserine lactones and biofilm formation in *Erwinia carotovora*, *Yersinia enterocolitica*, and *Aeromonas hydrophila*. *J. Agric. Food Chem.* 57 11186–11193. 10.1021/jf9029139 19950997

[B51] Van Delden C. Iglewski B. H. (1998). Cell-to-cell signaling and *Pseudomonas aeruginosa* infections. *Emerg. Infect. Dis.* 4 551–560. 9866731 10.3201/eid0404.980405 PMC2640238

[B52] Varga Z. G. Armada A. Cerca P. Amaral L. Mior Ahmad Subki M. A. Savka M. A. (2012). Inhibition of quorum sensing and efflux pump system by trifluoromethyl ketone proton pump inhibitors. *In Vivo.* 26 277–285. 22351670

[B53] Vattem D. A. Mihalik K. Crixell S. H. McLean R. J. C. (2007). Dietary phytochemicals as quorum sensing inhibitors. *Fitoterapia* 78 302–310. 10.1016/j.fitote.2007.03.009 17499938

[B54] Vu B. Chen M. Crawford R. J. Ivanova E. P. (2009). Bacterial extracellular polysaccharides involved in biofilm formation. *Molecules* 14 2535–2554. 10.3390/molecules14072535 19633622 PMC6254922

[B55] Wang W. Z. Sato T. Ito S. Kato N. Morohoshi T. Ikeda T. (2009). Inhibition of quorum sensing in gram-negative bacteria using AHL analogues and modified cyclodextrins. *J. Biosci. Bioeng.* 108 S38–S38. 10.1016/j.jbiosc.2013.01.022 23466297

[B56] Watnick P. I. Kolter R. (1999). Steps in the development of a *Vibrio cholerae* El Tor biofilm. *Mol. Microbiol.* 34 586–595. 10.1046/j.1365-2958.1999.01624.x 10564499 PMC2860543

[B57] Williams P. (2007). Quorum sensing, communication and cross-kingdom signalling in the bacterial world. *Microbiology* 153 3923–3938. 10.1099/mic.0.2007/012856-0 18048907

[B58] Winstanley C. Fothergill J. L. (2009). The role of quorum sensing in chronic cystic fibrosis *Pseudomonas aeruginosa* infections. *FEMS Microbiol. Lett.* 290 1–9. 10.1111/j.1574-6968.2008.01394.x 19016870

[B59] Zahin M. Ahmad I. Aqil F. (2016). Antioxidant and antimutagenic potential of *Psidium guajava* leaf extracts. *ıDrug Chem*. *Toxicol.* 2 1–8. 10.1080/01480545.2016.1188397 27268266

[B60] Zahin M. Aqil F. Husain F. M. Ahmad I. (2013). Antioxidant capacity and antimutagenic potential of *Murraya koenigii*. *Biomed. Res. Int.* 2013:263509 10.1155/2013/263509 PMC3703397 23853769

[B61] Zahin M. Hasan S. Aqil F. Khan M. S. A. Husain F. M. Ahmad I. (2010). Screening of certain medicinal plants from India for their anti-quorum sensing activity. *Indian J. Exp. Biol.* 48 1219–1224. 21250604

[B62] Zhao Z. G. Yan S. S. Yu Y. M. Mi N. Zhang L. X. Liu J. (2013). An aqueous extract of Yunnan baiyao inhibits the quorum-sensing-related virulence of *Pseudomonas aeruginosa*. *J. Microbiol.* 51 207–212. 10.1007/s12275-013-2595-x 23625222

[B63] Zhou L. M. Zheng H. D. Tang Y. D. Yu W. G. Gong Q. H. (2013). Eugenol inhibits quorum sensing at sub-inhibitory concentrations. *Biotechnol. Lett.* 35 631–637. 10.1007/s10529-012-1126-x 23264268

