# Supplementary material for: Leaf Extracts of Mangifera indica L. Inhibit Quorum Sensing – Regulated Production of Virulence Factors and Biofilm in Test Bacteria
Source: Front Microbiol. 2017 Apr 24;8:727. doi: 10.3389/fmicb.2017.00727 (PMC5402315; doi:10.3389/fmicb.2017.00727)
Supplement: Supplementary file 1 [file Table_1.DOCX]

**Supplementary figures**

**Figure S1.** Growth kinetics studies on a). *P. aeruginosa* PAO1 and b). *A. hydrophila* WAF38 in the absence and presence of sub-MICs of *M. indica* extract.

**Figure S2.** GC-MS chromatogram of methanol extract of *Mangifera indica* L.

**
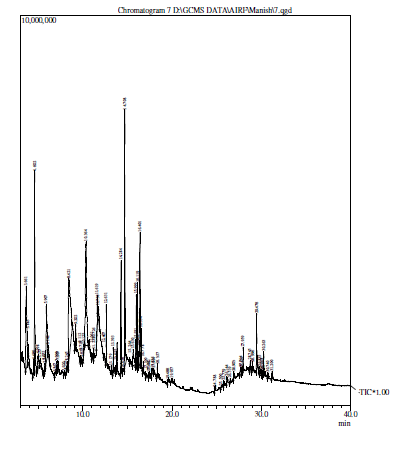
**

**Figure S3:** Structures of compounds as identified by GC-MS analysis

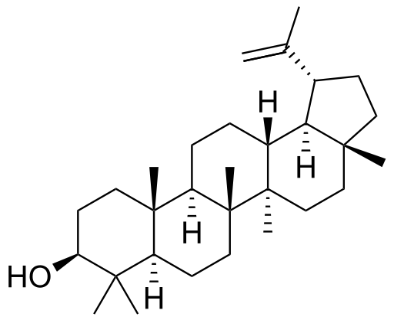
Lupeol
